# A Cladist is a systematist who seeks a natural classification: some comments on Quinn (2017)

**DOI:** 10.1007/s10539-018-9621-7

**Published:** 2018-03-28

**Authors:** David M. Williams, Malte C. Ebach

**Affiliations:** 1 Department of Life Sciences, The Natural History Museum, Cromwell Road, London, SW7 5BD UK; 2 Palaeontology, Geobiology and Earth Archives Research Centre (PANGEA), School of Biological, Earth and Environmental Sciences, UNSW Australia, Sydney, NSW 2052 Australia

**Keywords:** Artificial classification, Natural classification, Augustin P. de Candolle, Carl Linnaeus, Gareth Nelson, Systematics

## Abstract

In response to Quinn (Biol Philos, 2017. 10.1007/s10539-017-9577-z) we identify cladistics to be about natural classifications and their discovery and thereby propose to add an eighth cladistic definition to Quinn’s list, namely the systematist who seeks to discover natural classifications, regardless of their affiliation, theoretical or methodological justifications.

Derived from various permutations of phylogeny, biology, philosophy, methodology, sociology, loyalty etc., Aleta Quinn recently proposed “seven specific definitions that capture distinct contemporary uses” of cladistics (Quinn 2017, p. 1). Our own efforts, based on the same criteria, yielded a further seven, which we do not intend to bore our readers with here. We are sure more could be found and more people could be found who subscribe/correspond to them. Suffice to say, one might find definitions for anything—and in any case, Quinn was clear about her motives: “I do not intend to classify individuals, ideas, or research programs. Rather, I clarify distinct things that speakers mean by the term ‘cladist’” (Quinn 2017, p. 1). Depending on one’s outlook—philosopher, historian, biologist, even sociologist (Hull 1988)—the definitions might help progress their subject. As biologists, we found much to think about but rather than dissecting the minutiae, we seek to clarify by attempting to simplify.

We need first to dispense with one misconception. Quinn draws upon a commonly preconceived notion, namely that systematics requires evolution as a prior condition:^1^

“What that theoretical foundation may have been [in reference to de Candolle’s view on characters] is not relevant to my points about contemporary systematics, whose conceptual framework presupposes the concept of evolution” (Quinn 2017, footnote 11).

Consider the concept of a cladogram, which everyone might agree is a branching diagram commonly included as part of the results of a cladistic analysis. One might derive from this diagram which taxon is more closely related to itself than to any other. One might explain this relationship by common descent. The cladogram, however, need not be constructed with any evolutionary assumptions in mind; rather, the evolutionary assumptions serve to explain why one taxon is more closely related to itself than any other.

The search for a natural classification was established prior to the adoption of any theory of evolution. In fact Augustin P. de Candolle’s had a great deal to say on the matter, especially the differences between natural and artificial classifications (Candolle 1913). But de Candolle was working some time ago, so what, if anything, might be his relevance today? Methods of systematics change as time passes. But all methods find cladograms, in the sense that the results yield sets of relationships, either as a branching diagram or as a written classification. Regardless of method, which of these relationships might be considered to reflect something that actually exists, rather than a product (an artefact) of the method? How can any method achieve that without knowing the answer beforehand? Obviously it can’t. One might play around with simulation studies to judge the performance of any suite of methods, or one might delve into philosophy to create justification, but in the court of last resort all that remains are sets of cladograms that either agree or disagree to a greater or lesser extent in terms of common relationships found. That is, they agree in the cladistic parameter, the relationships specified—that the signal to noise ratio is working in our favour, as is evident from classifications of the past. Here we might argue that natural classification is the result derived from several cladograms, regardless as to how they were arrived at; artificial classifications are derived from a specific method, be that Wagner parsimony, UPGMA, maximum likelihood and so on, or from a specific source of data (DNA, ultrastructure, etc.), and so on. Why are these artificial? Because a method, any method, assumes the results that are required (the shortest tree; or the most similar taxa grouped together; or the most similar taxa grouped together via a weighted model of character change, etc.); for a data source, they assume those data are privileged over other data (DNA *must* be the source of ‘true’ relationships, etc.). Cladistics, in its most general sense, does not associate with any one method, or any one data source. It applies to sets of relationships—it *is* the set of relationships. This is effectively what de Candolle argued for, and has been the basis of systematics for decades, if not centuries:

“For the last 50 years and more—even now continuing into the realm of nomenclature—in the name of the modern and the new, Visionaries aim, as it were, to confine the past to a dustbin of history, and to bolt and lock the lid upon it. As if without it, we be in some way better, even born again more whole-some; as if Carl Linnaeus really were among the last of the Ancients, and not, rightly, the first of the moderns, and so related to us—of a group inclusive of us” (*Annual Review of the Linnean Society*, 2001).

These words, not readily accessible, were spoken by Gareth Nelson after receiving the Linnean Gold Medal and re-cast above as part of the 2001 *Annual Review of the Linnean Society*, London. Linnaeus as the first of the moderns? Among other matters, Linnaeus spoke of the differences between artificial and natural classification, a subject taken up and developed by de Candolle (1913). One might cast that debate in very simple terms: artificial classifications are found by imposition, natural classification is discovered. Imposition implies some method or motivation to erect a particular classification, such as a field guide or handbook for identifying specimens—today it is more likely those would be websites, or online interactive guides. There is nothing wrong with artificial classifications. We both use them all the time, almost every day (https://www.trilobites.info/; http://naturalhistory.museumwales.ac.uk/diatoms/). But whatever merits they have, and there are many, they are created by acts of imposition. We ask our readers, then, if they would consider analysis of some data with one or another statistical program, or with one or another parsimony program, or with one or another phenetic program, whether this is an act of imposition or an act of discovery? We see it as an act of imposition. How could it be otherwise? Cladistics, then, *is about discovery*, about finding repeating patterns, finding the same relationships, finding relationships that are not method dependent, finding relationships that are reflections of the world as it is:

“What, then, of cladistics in relation to the history of systematics? If cladistics is merely a restatement of the principles of natural classification, why has cladistics been the subject of argument? I suspect that the argument is largely misplaced, and that the misplacement stems, as de Candolle suggests, from confounding the goals of artificial and natural systems” (Nelson 1979, p. 20).

For us, cladistics is about natural classifications and their discovery, an activity that occurs with or without “knowledge of process”. Look in museums, herbaria, universities and other institutions that still hire systematists and you will see:

Cladist (viii): A cladist is a systematist who seeks to discover natural classifications.

## Footnotes

1 The misconception that systematics “presupposes the concept of evolution” has been discussed in detail by Brady (1982) in response to Beatty (1982), Ghiselin (1969, 1974), Hull (1976, 1978) and Laudan (1977).
